# A Chinese question and answer system for liver cancer based on knowledge graph and large language mode

**DOI:** 10.3389/frai.2025.1663891

**Published:** 2025-10-10

**Authors:** Haoqi Wu, Min Zhang, Hailing Wang, Xiaoyan Jiang, Yongbin Gao, Rong Huang, Zhijun Fang, Xiaojun Hu, Yingfang Fan

**Affiliations:** ^1^School of Electronic and Electrical Engineering, Shanghai University Of Engineering Science, Shanghai, China; ^2^Department of Endocrinology, Ninth Hospital of Xi'an, Xian, China; ^3^The Hepatobiliary Surgery, Third Affiliated Hospital, Southern Medical University, Guangzhou, China

**Keywords:** large language model, question and answer system, liver cancer, knowledge graph, data integration

## Abstract

**Introduction:**

The liver cancer question-and-answer (Q&A) system is primarily intended to help patients access disease-related information more conveniently. However, there is currently no Q&A system specifically developed for liver cancer. Additionally, most existing Q&A systems lack real clinical data and have limited capability in understanding Chinese questions.

**Methods:**

This paper proposes a Chinese liver cancer question-answering system based on knowledge graphs and Large Language Models (LLMs). To unify information from diverse sources, the system employs a knowledge graph to store entities and inter-entity relationships extracted from patients' clinical electronic medical records and the professional medical website xywy.com, which serves as the foundation for the system's responses. Specifically, ChatGLM3.5 is utilized to extract entity information from questions, while BERT is applied to understand users' intent. Subsequently, the system retrieves corresponding information from the knowledge graph. Finally, the retrieved information is integrated, and a natural language response is generated as the answer to the question.

**Results:**

The experimental results indicate that in terms of intent classification, our system achieves a precision of 92.34%, representing an improvement of 1.38% over the BERT model and 4.32% over the GEBERT model. In terms of response relevance, the system's outputs are more aligned with patients' daily speech patterns and exhibit higher relevance to the target questions.

**Discussion:**

In conclusion, the improved method significantly enhances the usefulness and reliability of the liver cancer Q&A system.

## 1 Introduction

Liver cancer, a malignant tumor that severely endangers the lives and health of Chinese people, presents a worrying picture in terms of incidence and mortality. According to data released by the National Cancer Center of China, the number of new liver cancer cases ranks fourth in the country, while the number of deaths caused by this malignant tumor ranks second nationwide (Chinese Society of Liver Cancer, 2025). Therefore, providing more effective medical services to these patients so that they can quickly retrieve information about the disease has become a critical issue. With the popularity of the Internet, people increasingly rely on web search to seek answers to their medical questions. In this context, personalized Q&A systems have emerged as a more effective method for retrieving health-related information (Luo et al., 2022). These systems aim to offer more convenient access to medical knowledge, with key technologies involving knowledge storage and question parsing. However, as a cancer type with a high mortality rate in China, liver cancer cannot be provided with better Q&A services for patients by existing medical Q&A systems, such as general models based on electronic health records (Yang X. et al., 2022) and open-domain medical reasoning models (Liévin et al., 2024), which are not designed for clinical information of liver cancer.

Personalized medical Q&A systems (Oduro-Afriyie and Jamil, 2023; Cui et al., 2017) have gradually become part of people's daily lives as they provide tailored responses based on the patient's specific conditions, thereby improving patient outcomes. Several techniques, including machine learning, natural language processing, and data mining, have been utilized to enhance system performance (Chen et al., 2022; Lu et al., 2022). However, these methods often require retraining to adapt to new data and usage environments, limiting their ability to cope with dynamically changing information.

Traditional Q&A systems rely heavily on publicly available unstructured data, increasing the complexity of answer retrieval. The introduction of knowledge graphs provides a structured approach to querying large amounts of data efficiently, thereby improving answer retrieval speed in Q&A systems (Li et al., 2020). Knowledge graph-based Q&A systems have gained significant attention for their ability to facilitate data access (Lan et al., 2021). Although personalized knowledge graphs have been validated for their value in chronic diseases (Gentile et al., 2019), thyroid diseases (Chai, 2020), and herbal medicine information (Yang Y. et al., 2022), there remains a significant gap in the field of liver cancer. In addition, existing medical Q&A systems primarily use publicly available data and seldom incorporate private information such as electronic medical records (Oduro-Afriyie and Jamil, 2023). This limits their ability to provide personalized recommendations. Integrating patients' medical records into knowledge graphs while ensuring data security and preventing privacy breaches remains a significant challenge.

Knowledge graphs have been widely applied in various domains and have proven effective in medical Q&A systems (Li et al., 2023, 2024). However, most existing knowledge graphs are constructed using publicly available data, neglecting valuable patient-specific information such as medical history (Dutt et al., 2022; Gentile et al., 2019; Gyrard et al., 2018; Shirai et al., 2021). Moreover, responses generated from these systems tend to be mechanistic and lack fluency. To address these shortcomings, our proposed system integrates both public and private medical data to offer personalized healthcare Q&A services. Furthermore, by leveraging natural language processing techniques and large language models (LLMs), we enhance the readability and naturalness of the generated responses.

With the widespread adoption of LLMs in various fields, they have demonstrated strong generalization abilities and have been applied to diverse research areas. Compared with traditional machine learning methods, LLMs offer better interpretability and increasingly advanced natural language processing capabilities. In recent years, LLMs such as GPT-3 (Brown et al., 2020), BERT (Devlin et al., 2019), and ChatGLM3.5 (Du et al., 2022) have achieved impressive results in healthcare applications, including medical Q&A (Chowdhery et al., 2024; Guu et al., 2020; Liévin et al., 2024; Yang X. et al., 2022). However, although current medical question-and-answer systems perform excellently in general disease Q&A, due to the uniqueness of different diseases, the accurate understanding of individual patients' conditions by general medical question-and-answer systems may lead to inaccurate or misleading suggestions (Maynez et al., 2020). Despite their advantages in extracting knowledge and providing insightful responses, LLMs lack awareness of individual patient conditions, which can lead to inaccurate or misleading recommendations (Maynez et al., 2020). Additionally, directly providing patient data to LLMs poses significant privacy risks. Our proposed system addresses these challenges by incorporating LLMs' natural language processing capabilities while integrating electronic medical records in a privacy-preserving manner.

To overcome the limitations of existing methods, this paper proposes a Chinese Q&A system for liver cancer patients that combines Large Language models (LLMs) with knowledge graphs. The system utilizes electronic health records and various disease-related data as knowledge sources while anonymizing personal identity information to prevent data leakage. The system uses LLMs to extract entity information from user queries and employs BERT models to analyze user intent, thereby enhancing its ability to understand user queries. It can more accurately retrieve relevant information from the knowledge graph. Finally, leveraging the advanced language processing capabilities of ChatGLM3.5, the system integrates the retrieved information to improve the coherence and fluency of responses. This system provides liver cancer patients with an efficient tool to consult about their condition and receive simple, reliable advice on treatment and lifestyle adjustments.

## 2 Materials and methods

**Figure 1:**
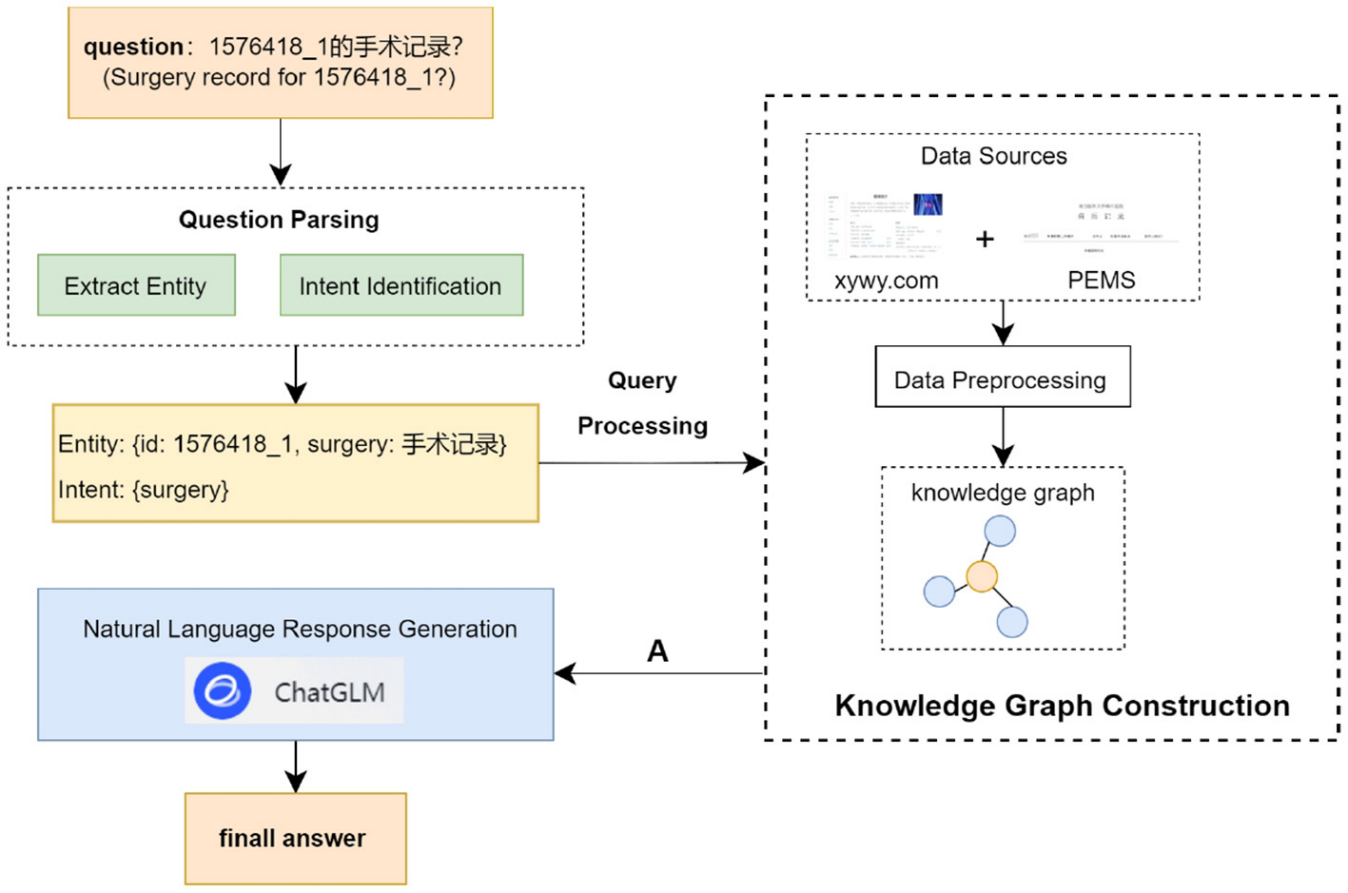
Overall architecture of personalized Q&A system based on the knowledge graph.

In this section, we introduce the construction of a liver cancer knowledge graph and the architecture of a Chinese liver cancer Q&A system. The overall structure of the Chinese liver cancer Q&A system is shown in Figure 1. It is mainly driven by four functions: liver cancer knowledge graph, question parsing, query processing and natural language response generation.

### 2.1 Knowledge graph construction

This section describes the data sources for the Liver Cancer Knowledge Graph, and how to process the data for knowledge graph construction.

#### 2.1.1 Data sources

Data from two main sources is integrated: patient electronic medical records (PEMRs) provided by Zhujiang Hospital of Southern Medical University, and disease information extracted from xywy.com.

From 2010 to 2022, Zhujiang Hospital of Southern Medical University provided 144 PEMRs of HCC that were confirmed by surgery or puncture pathology. The PEMRs contain the patient's personal information, the patient's admission record (patient's personal information, complaints, doctor's examination results, etc.), the disease course record (illness, examination records, etc.), the surgery record (surgery time, blood transfusion, name of the surgery, etc.), and the discharge summary (the patient's overall situation from admission to discharge). The PEMRs are a complete and accurate record of the patient's illness, and careful attention should be taken to safeguard the patient's privacy and prevent their private information from being released during processing.

The description of disease information on xywy.com involves many aspects, including basic information about the disease (name of the disease, causes of its onset, clinical manifestations, etc.), diagnosis and examination (diagnostic methods such as laboratory tests, imaging tests; examination items such as blood tests, urine tests, etc.), treatment and rehabilitation (treatment methods such as medication, surgical treatments, etc.), rehabilitation recommendations (dietary adjustments, lifestyle changes, etc.), prevention and health care (preventive measures and health care knowledge of the disease), and prevention and health care (disease prevention measures and health care knowledge).

#### 2.1.2 Data preprocessing

Raw unstructured medical records and semi-structured website data are converted into structured form for knowledge graph construction using different approaches, respectively.

1. PEMRs preprocessing

Patient's electronic medical records are stored in the form of unstructured text, to store them in the form of knowledge graph, they need to be processed into a specific structured form for storage. In order to improve the processing speed and quality, we construct a named entity recognition dataset based on patient's EHRs for model training so as to batch process PEMRs.

The entity annotation tool YEDDA is utilized to annotate eight types of data for each type of entity in the PEMR. Among them, ID is used to uniquely label the patient; body is the patient's self-reported physical condition; bodyexam is the patient's physical examination result; symptom is the patient's symptom; disease is the patient's acquired disease; result is the test result of the examination item; surgery is the patient's surgical record; and cure is the patient's disease treatment. Using this method, a total of 44 PEMRs of patients were processed and labeled in BIO format. And they are divided into training set, validation set and test set according to the ratio of 8:1:1, which is used as the basis for training and evaluation of named entity recognition model. Thus, batch processing of PEMRs was realized.

We use RoBERT+BiLSTM+CRF to construct the named entity recognition model. The model's hyperparameters are set as follows: batch-size is 40, epoch is 20, learning rate is 10^−5^, LSTM hidden layer size is 128, and the maximum sentence length is 50. RoBERT model is a pre-training model more similar to the BERT model. A dynamic masking mechanism is used, especially in the Chinese context, which employs the use of word segmentation processing, which facilitates the model to capture more word-level information. BiLSTM-CRF model, where BiLSTM is used to extract high-level feature representations of the text, while the CRF layer is responsible for selecting the most appropriate labels for the whole sequence based on these features. This combination takes full advantage of BiLSTM's strength in capturing long-distance dependencies and CRF's power in sequence modeling, making the BiLSTM-CRF model excel in a variety of NER tasks.

The named entity recognition model enables rapid completion of the processing of the remaining patient's electronic medical records. After extracting the data information, we perform ID anonymization through a hash function (SHA-256) to map the original patient ID to an irreversible string, ensuring that the anonymized ID cannot be reversed to the original identifier while maintaining uniqueness for distinguishing different patients. The processed records, with anonymized IDs, are stored in json format for subsequent knowledge graph construction.

In handling patients' electronic medical records, we strictly adhere to the ethical guidelines for medical research. All patients included in the study signed an informed consent form upon admission, agreeing to the use of their clinical data for relevant clinical research. For data involving patients' personal information, all such data undergoes anonymization before being used in knowledge graph construction; electronic medical record materials are used exclusively for this study and will never be utilized for any purposes unrelated to the research.

2. Publicly website database preprocessing

Disease data in http://xywy.com is semi-structured text and medical data is crawled using crawler technology. Through in-depth analysis of the html text information in the crawled webpage, entity information such as medicine, symptom and disease is extracted and processed into structured data. Medical text involves a large number of proper nouns, which need to be further sliced and diced. Medical text involves a large number of proper nouns, which requires further slicing and dicing of the text content. Taking the content corresponding to the complications of “cold” as an example, the corresponding text before word splitting is “rhinitis, otitis media, tonsillitis”, and according to the maximum bidirectional word splitting algorithm, a method that combines forward maximum matching and backward maximum matching to segment text, first performing forward maximum matching from the start of the text, then backward maximum matching from the end, and determining the optimal segmentation result by comparing and integrating the two results based on predefined rules (Gai et al., 2014). It is necessary to slice the text again to extract a single noun. The processed data is stored as structured data in json format for subsequent knowledge graph construction, which contains 7 categories of data examination, department, disease, drug, food, manufacturer and symptom.

3. Data specification

In addition, there may be ambiguities such as irregular or inaccurate naming in the data from electronic medical records and websites. To eliminate such ambiguities, this study will also use the Common Clinical Medical Terms (2023 Edition). It is a professional medical reference book designed to provide accurate and systematic medical terminology references for medical professionals, students, and workers in related medical and health fields. This book contains a large number of medical terms, covering various branches of clinical medicine, such as internal medicine, surgery, obstetrics and gynecology, pediatrics, emergency medicine, preventive medicine, etc.

#### 2.1.3 Knowledge graph

Based on the processed data in JSON format, we extract triple information and import it into the graph database Neo4j. To ensure the comprehensiveness and accuracy of the knowledge graph, we perform knowledge fusion to address issues such as non-standard entity naming and mismatches between electronic medical records (EMRs) and website data. Specifically, we use the TF-IDF algorithm to calculate the cosine similarity between entities in EMRs and those in the website, thereby achieving entity alignment. For entities that cannot be directly matched (“gallbladder stones with chronic cholecystitis” in EMRs), we conduct word segmentation on them according to Common Clinical Medical Terms (2023 Edition), splitting them into standard entities (“gallbladder stones” and “chronic cholecystitis”) for normalization. After alignment, we remove duplicate nodes and establish new relationships to integrate entities from these two sources.

The final knowledge graph encompasses 13 entity types and 17 inter-entity relationships (Table 1). Relationship types include:

**Table 1:** Knowledge graph data information.

**Entity type**	**Number**	**Relationship type**	**Number**
ID	144	Recommand_eat	40,221
Disease	9052	No_eat	22,247
Check	3353	Do_eat	22,238
Body	147	Belongs_to	8,844
Bodyexam	114	Common_drug	14,649
Cure	177	Drugs_of	17,315
Department	54	Recommand_drug	59,467
Drug	3828	Has_symptom	5,998
Food	4870	Need_check	39,422
Producer	17201	Acompany_with	12,029
Result	324	Rel_body	240
Surgery	341	Rel_symptom	585
Symptom	6464	Rel_bodyexam	1,536
		Rel_disease	694
		Rel_result	377
		Rel_surgery	752
		Rel_cure	288
Total number of entity	46,069	total number of relationship	246,904

(1) Disease-food: recommand_eat (recommended foods to eat), no_eat (foods to avoid), do_eat (suitable foods);

(2) Disease-related: belongs_to (affiliated department), common_drug (commonly used drugs), drugs_of (related drugs), recommand_drug (recommended drugs), has_symptom (symptom manifestations), need_check (required examinations), acompany_with (accompanying symptoms);

(3) Patient records: rel_body (self - reported condition), rel_symptom (symptom - related), rel_bodyexam (physical examination results), rel_disease (related diseases), rel_result (test results), rel_surgery (surgery - related), rel_cure (treatment methods)

### 2.2 Knowledge graph and LLM based Q&A system

#### 2.2.1 Question parsing

1. Extract Entity

It is the key technology for the implementation of personalized Q&A system. Determining the central entity in the question sentence is the core step to realize the query, and the answer to the question is directly or indirectly related to the central entity in the question sentence. Therefore, after determining the central entity contained in the question sentence, the answer to the question can be queried according to the connection relationship between the entities.

This paper designs a named entity recognition module based on ChatGLM, which extracts entities from questions through an explicit function call mechanism: we predefine an external entity extraction function and pass it to ChatGLM. Among them, the tools parameter specifically specifies the types of entities to be extracted (as shown in Table 2) and their constraints through description which defines the detailed characteristics of entities and type which defines entity category labels. When processing a question, ChatGLM will call this predefined function according to instructions and output the extracted entities in a structured format that strictly conforms to the specified entity types and constraints. Compared with the extraction method directly based on prompt words, it can enforce standardized output. In addition, the explicit constraint definitions in the function will guide the model to focus on the description and type of entities, reducing the misrecognition of irrelevant entities.

**Table 2:** Tools parameter settings.

**Name**	**Description**
Get_people_answer	Answer a variety of medical-related questions based on Patient questions
Id	Patient id number
Body	Patient's physical condition or complaint
Bodyexam	Patient checkup information
Check	Patient checks or diseases requiring checks
Cure	Patient cure or cure for a disease
Disease	Name of the disease or disease that the patient has
Result	Patient results
Surgery	Patient surgery records
Department	Department of medicine
Drug	Commonly used drugs
Food	Name of food
Symptom	Symptoms associated with the patient or disease
Deny	Negative word

**Figure 2:**
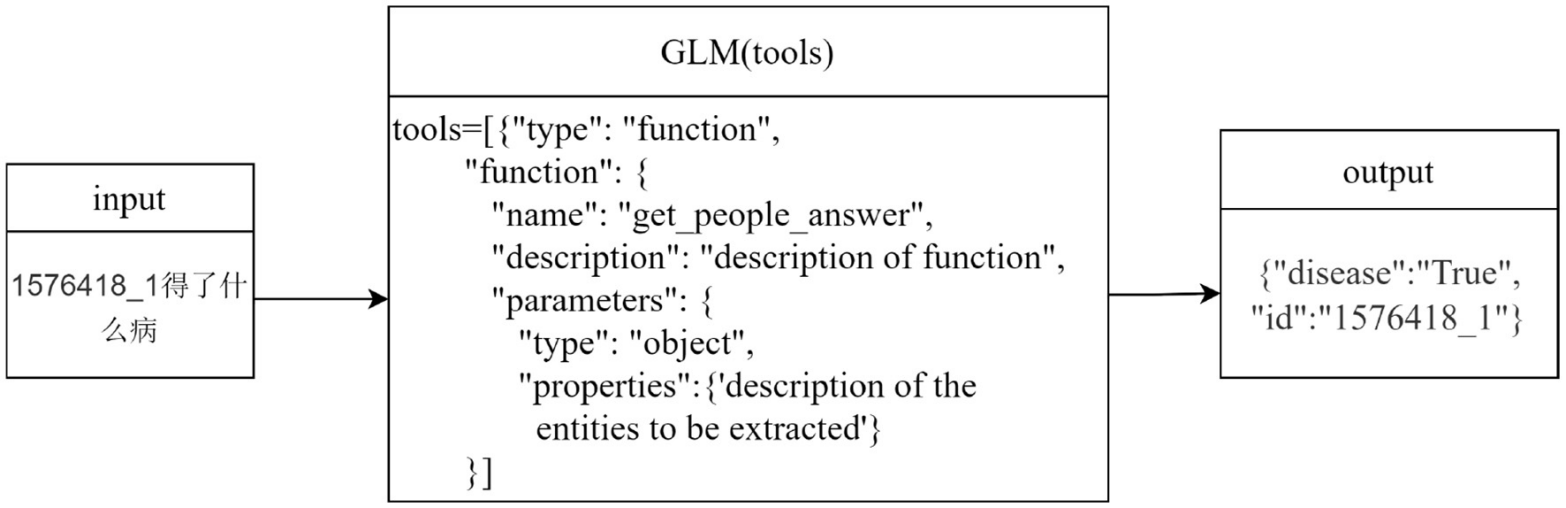
Named entity extraction.

Take the question “What disease does 1576418_1 have?” as an example, as shown in Figure 2. After the user inputs the question, it is matched with the descriptions of the preset parameters in the tools in the ChatGLM. “1576418_1” is matched with the parameter “id” described as the patient id; “What disease” matches the parameter “disease” described as the name of the patient's disease. Thus, the content of the problem that matches the parameter description is extracted, and using this way of extracting entities, not only entities with actual meanings in the problem can be identified, but also questionable words that are associated with the parameter description can be identified.

2. Intent Identification

This system is a medical-based Q&A system, which saves only the knowledge about medical treatment in the knowledge graph, in order to improve the understanding of the user's intent and to limit the scope of questions answered by the ChatGLM. Therefore, this system adds the BERT model to identify the user's intention while filtering questions that are not related to medical care.

**Figure 3:**
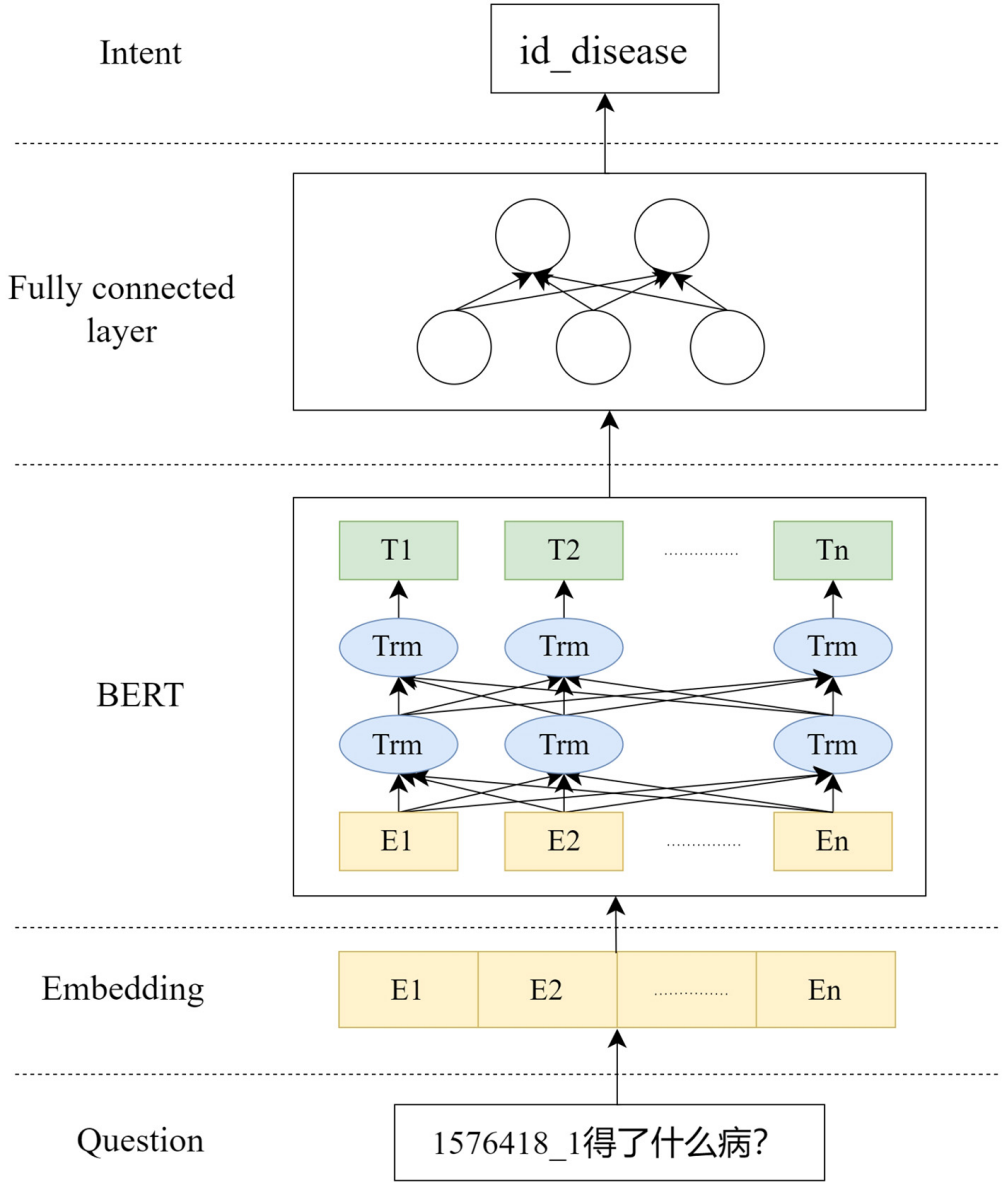
Intent identification module.

The training process is shown in Figure 3, where the training data is input into BERT, and then the result of BERT is input into the fully connected layer to get the pair classification result intent. According to the change of the number of labels in the dataset, the training only needs to update the parameters of the fully connected layer. Typically, using BERT for the intention recognition task, the classification word vector H of the BERT result is used to make a simple classifier based on softmax to predict the probability of the label L of the category:


(1) $$\begin{array}{lll} P\left(L|H\right) & = & softmax\left(WH\right) \end{array}$$

W is the parameter matrix for the classification task, which is ultimately fine-tuned by maximizing the logarithmic probability of labels for all parameters in BERT and W. Modify it to get the probability of each label using the fully connected layer:


(2) $$\begin{array}{lll} P\left(L|H\right) & = & FC\left(H\right) \end{array}$$

The output dimension is the number of label categories, in this case it is a multiple classification task where the label with the highest probability is finally selected as the result of classification. In the medical Q&A domain, the intent is the patient's intention, and by understanding the intention, the scope of the question answer is de-qualified and along with the result of named entity extraction, it is judged whether a professional answer can be given or not.

To quickly construct diverse question types under different intents, we leverage ChatGLM3.5 to achieve rapid generation of sentence types. Specifically, we design prompts to guide the model in generating the required corpus. For example: “Assume you are an experienced deep learning trainer and now want to build a batch of datasets for intent recognition classification. The patient's questions should be as varied and colloquial as possible, with sentences of varying lengths and avoiding repetitive structures. Specific medical entities involved in the corpus templates should be replaced with placeholders; for instance, disease names are replaced with [disease]. List the questions directly in items, try to include as many as possible, with at least 100 questions. Each sentence must end with a question mark. Example: What is [disease]?” Using the above method, we can quickly obtain intent-based question templates (as shown in Table 3). These intent categories correspond to the 25 inter-entity relationship types in the knowledge graph, covering multiple dimensions such as food, drugs, symptom prevention, treatment cycle, susceptible groups, disease-related examination items, affiliated departments, and complications. They match the relationship types in the predefined template library of the query processing module, providing a rich and contextually relevant corpus foundation for the training of the intent recognition model.

**Table 3:** Medical Intent Form.

**Intent name**	**Example**
Food	I have [disease], can I drink [food]?
Medicine	Please recommend some effective medicines for [disease]?
Symptom Prevention	How to prevent the occurrence of [disease]?
Treatment Cycle	If I have [disease], how long do I need treatment?
Affiliated Department	Which department does [disease] belong to?

#### 2.2.2 Query processing

The patient input question has been analyzed for user intent after the question parsing module, and the query statement needs to be constructed based on the intent has been searched for the answer to the question from the constructed knowledge graph. This system constructs the query statement by template matching and then searches for the answer in the knowledge graph. The specific process is as follows:

1. Match the corresponding Cipher query statement templates from a predefined template library. This template library contains 25 templates, categorized by inter-entity relationships and entity attributes. For example, for the “Disease-Food” entity pair and the “recommand_eat” relationship, the template is MATCH (m:disease)-[r:recommand_eat] → (n:food) where m.name = “Entity” return m.name, r.name, n.name;
2. Using the extracted entity names to populate the placeholders in the templates so that the query statement is transformed into a complete query statement;
3. Retrieving the answer to a question through the neo4j query interface;
4. Integrate the node information in the graph through answer templates and return it to ChatGLM for further inference and analysis. If no matching data is found in the knowledge graph, no data will be returned to ChatGLM.

The system writes different templates to map user interrogations into query statements for the graph database based on predefined intents and entities. The query statement construction and answer generation is performed once for each intent and entity individually, before finally returning all the generated answers to the ChatGLM.

#### 2.2.3 Natural language response generation

The content returned in Section 2.2.2 is a set A from the knowledge graph entities and relations that represent the concepts and actions used to answer the user's question. We view A as a set of scrambled phrases that make up the answer text, and our goal is to compose the answer text by reconstructing them. This takes into account the connectivity between the words. For example, the question text “What did Ming do after he woke up?", and set A “eat breakfast, wash up.” Based on the results of the query, human beings rearrange them and add missing associative words, subjects, etc. such as “he, first, after,” so as to form a complete statement “he washed up first and then ate breakfast.” In line with this approach, ChatGLM is guided to operate through prompt settings, enabling it to match the content in set A with appropriate natural language expressions based on the semantics of patients' questions. Meanwhile, since ChatGLM cannot obtain knowledge graph information beyond set A, if it is allowed to call its own knowledge base to search for answers, it may generate complex and diverse results that deviate from the limitations of the knowledge graph. Therefore, specific constraints are added to the prompts to prohibit the model from using any knowledge outside set A, so as to ensure that the final answer is generated entirely based on the information in set A.

Under this mechanism, ChatGLM is restricted to the role of a natural language generator. It only focuses on the information returned by the knowledge graph and reorganizes scattered phrases into complete expressions that conform to the habits of daily language. When the knowledge graph returns no information (that is, set A is empty), ChatGLM will directly prompt the user with “No relevant information found” to ensure the authenticity and reliability of the answer.

## 3 Results and discussion

This section assesses the validity of the proposed system in two parts: (1) performance evaluation; (2) different model answering effects.

### 3.1 Performance evaluation

To validate the effectiveness of the question parsing module, i.e., to recognize the intention expressed by the patient in the input discourse and the entity information in it, examples of different types of intentions are needed, each of which contains a different type of questioning. In order to quickly construct the types of questioning under different intentions, we let ChatGLM3.5 construct a batch of datasets for performing intention recognition classification by setting specific prompts.

We compared our method with BERT and gemini-1.0-pro+BERT (GEBERT). The same parameter metrics are used in different methods to evaluate their effectiveness in intent recognition. From the results in Table 4, the method proposed in this paper is improved in all the metrics, indicating that it can understand the user's intention more effectively. This is because the method proposed in this paper combines the ability to understand the semantic information of BERT and the entity extraction ability of ChatGLM, which enables it to understand the patient's intention more accurately.

**Table 4:** Results of comparative experiments.

	**Bert**	**GEBERT**	**Ours**
Precision	0.9096	0.8802	**0.9234**
Recall	0.9056	0.8542	**0.9158**
F1	0.9048	0.8553	**0.9196**

Bold values indicate the best results.

To objectively verify the question-answering performance of the system, we had the system answer single-choice questions related to liver cancer expertise to conduct an objective evaluation of its performance. We compared our proposed method with ChatGLM and gemini-1.0-pro+BERT (GEBERT). In this experiment, 100 single-choice questions with different difficulty levels were designed, and the accuracy of the question settings was verified by professional doctors. These questions were classified into 34 single-hop questions (SQ), 33 double-hop questions (DQ), and 33 multi-hop questions (MQ).

The classification criteria are as follows: Single-hop questions (SQ) involve direct retrieval of a single entity or relationship (e.g., “Which of the following is a common symptom of liver cancer?"); Double-hop questions (DQ) require the integration of multiple related entities or one reasoning jump (e.g., “Which examinations are typically required for diagnosing early liver cancer?"); Multi-hop questions (MQ) involve multi-step reasoning, complex relationship chains, or high-level knowledge in the domain (e.g., “What is the mechanism by which drug X inhibits the proliferation of liver cancer cells?").

Different models were allowed to answer these questions to calculate scores to evaluate the objective performance of the system. The results of the experiment are shown in Table 5, where the numbers in parentheses indicate the number of questions answered correctly and the accuracy rate is the number of questions answered correctly divided by the total number of questions.

**Table 5:** Scoring of multiple choice questions.

	**Accuracy**			
	**SQ**	**DQ**	**MQ**	**ALL**
ChatGLM	0.76	0.64	0.55	0.65
	−26	−21	−18	−65
GEBERT	0.79	0.76	0.61	0.72
	−27	−25	−20	−72
Ours	0.85	0.73	0.64	0.74
	−29	−24	−21	74

As shown in Table 5, the response accuracy of the models gradually decreases as the difficulty of the questions increases. The experimental results indicate that ChatGLM performs relatively weakly overall, with an accuracy of 0.55 on multi-hop questions (MQ), showing poor performance in handling more difficult problems. GEBERT performs slightly better than ChatGLM and has relatively good stability. Our method performs better across all question types. Although its accuracy on double-hop questions (DQ) is slightly lower than that of GEBERT, the proposed method still maintains a high accuracy rate, with an overall accuracy of 0.74, which is better than both ChatGLM and GEBERT. In summary, our method outperforms the other two models in terms of answering accuracy, demonstrating its effectiveness and advantages in question-answering tasks.

In order to validate the functionality of different modules of the system, ablation experiments are conducted here to validate the functionality of two modules, namely, question parsing and natural language generation. For the question parsing module, its role is to categorize the questions and extract the relevant information from the database, which is required to analyze the performance of the module using the problem dataset. For the natural language generation module, the essence of which is to generate more complete questions and answers from the database, the merits of the answer text cannot be judged by metrics, which are analyzed separately through Section 3.2. For the query processing module, the main function is to extract knowledge from the database and transfer it to the natural language generation module, testing only the effect of not including the database.

As shown in Table 6, after removing the question parsing module, although the system can still analyze questions based on prompt statements, its accuracy decreases by 3.24% and the F1 score decreases by 2.32%. Its performance is significantly lower than that of the system with the module retained, which verifies the necessity and effectiveness of the question parsing module. Removing the database has no impact on the answering of single-hop questions; however, its impact on double-hop questions and multi-hop questions is relatively obvious. The accuracy of double-hop questions decreases by 8.22%, and that of multi-hop questions decreases by 18.75%. This indicates that injecting relevant knowledge through the database can assist large language models in improving the answering accuracy of complex reasoning tasks, thereby verifying the rationality of adding the database.

**Table 6:** Results of ablation experiment.

	**Accuracy**		
	**Precision**	**Recall**	**F1**
Ours	0.9234	0.9158	0.9196
Ours w/o question parsing	0.8935	0.9013	0.8983
	SQ	DQ	MQ
Ours	0.85	0.73	0.64
Ours w/o dataset	0.85	0.67	0.52
	−29	−24	−21

### 3.2 Different model answering effects

In this paper, some natural question sentences are constructed to test the answering effect of the system. After inputting the interrogative sentences, the system is tested to be able to answer whether it can return the expected answers in line with the expected answers, and it can also further test the effect of the interrogative sentence parsing module, and some of the test results are shown in Table 7.

**Table 7:** Personalized Q&A system effect test.

(Q1) User:
1576418_1 得了什么病？
(Diseases for 1576418_1?)
(A1)
BERT: 1576418_1病人的疾病有:肝囊肿；左肝原发性肝癌；右侧胸腔积液；乙肝病毒携带者；肝脏占位；肝炎后肝硬化
(1576418_1 Patient's diseases include: liver cyst; primary hepatocellular carcinoma of the left liver; pleural effusion on the right side; hepatitis B virus carrier; liver occupancy; cirrhosis of the liver after hepatitis)
GEBERT: 肝囊肿；左肝原发性肝癌；右侧胸腔积液；乙肝病毒携带者；肝脏占位；肝炎后肝硬化
(liver cyst; primary hepatocellular carcinoma of the left liver; pleural effusion on the right side; hepatitis B virus carrier; liver occupancy; cirrhosis of the liver after hepatitis)
Ours: 病人1576418_1的诊断包括以下疾病:肝囊肿,肝炎后肝硬化,右侧胸腔积液,左肝原发性肝癌,乙肝病毒携带者,肝脏占位。这些疾病需要综合评估和治疗。
(Patient 1576418_1's diagnoses include the following diseases: hepatic cyst, post hepatitis cirrhosis, right pleural effusion, primary hepatocellular carcinoma of the left liver, hepatitis B carrier, and liver occupancy. These disorders require comprehensive evaluation and treatment.)
(Q2) User:
1576418_1的手术记录?
(Surgery record for 1576418_1?)
(A2)
BERT: 1576418_1病人的手术记录有:腹腔镜下探查中转开腹左肝外叶肝癌切除术；输血量:0ml；15:30；12:17
(Patient 1576418_1's surgical records include: laparoscopic exploration intermediate to open left extrahepatic lobe hepatectomy for hepatocellular carcinoma; blood transfusion: 0 ml; 15:30; 12:17)
GEBERT: 手术时间:12:17-15:30,手术名称:腹腔镜下探查中转开腹左肝外叶肝癌切除术,输血量:0ml
(Surgery time: 12:17-15:30, surgery name: Laparoscopic exploratory intermediate open left hepatic exophytic hepatectomy for hepatocellular carcinoma, blood transfusion: 0 ml)
Ours: 尊敬的患者,关于您询问的医疗记录,以下是 1576418_1 病人的手术记录摘要:手术类型:腹腔镜下探查中转开腹左肝外叶肝癌切除术,输血量:0 毫升,手术开始时间:12:17,手术结束时间:15:30。如果您还有任何问题或需要进一步的医疗信息,请联系您的医疗服务提供者。
(Dear Patient, regarding your enquiry about the medical record, the following is a summary of the surgical record for patient 1576418_1: Surgery type: laparoscopic exploratory intermediate open resection of hepatocellular carcinoma of the outer lobe of the left liver, blood transfusion: 0 ml, start time of the surgery: 12:17, end time of the surgery: 15:30. If you have any questions or need further medical information, please contact your healthcare provider.)
(Q3) User:
1576418_1所得疾病有什么并发症
(What are the complications of the disease 1576418_1 has?)
(A3)
BERT: 1576418_1病人的疾病有:肝囊肿；左肝原发性肝癌；右侧胸腔积液；乙肝病毒携带者；肝脏占位；肝炎后肝硬化
(1576418_1 Patient's diseases include: liver cyst; primary hepatocellular carcinoma of the left liver; pleural effusion on the right side; hepatitis B virus carrier; liver occupancy; cirrhosis of the liver after hepatitis)
GEBERT: 经过信息查询,您的疾病信息如下:肝囊肿,左肝原发性肝癌,右侧胸腔积液,乙肝病毒携带者,肝脏占位,肝炎后肝硬化。需要注意的是,我查询到的信息中没有提及您所患疾病的并发症。
(As a result of the information query, your disease information is as follows: liver cyst, primary hepatocellular carcinoma of the left liver, pleural effusion on the right side, hepatitis B virus carrier, liver occupancy, and cirrhosis of the liver after hepatitis. It is important to note that the information I have queried does not mention the complications of your disease.)
Ours: 病患1576418_1所患的疾病包括肝囊肿、肝炎后肝硬化、右侧胸腔积液、左肝原发性肝癌、乙肝病毒携带以及肝脏占位。这些病情可能导致的并发症包括但不限于以下几种:腹水,消化道出血,肝性脑病,肝肾综合征,肝肺综合征,更容易感染其他病毒或细菌,容易疲劳、黄疸（皮肤、眼睛、尿液变黄）等,脾功能亢进。请注意,以上信息仅供参考,具体的治疗方案和并发症的风险需要结合患者的具体情况和医生的专业建议。建议病患及时就医,接受专业的诊断和治疗。
(Diseases suffered by patient 1576418_1 include hepatic cysts, post hepatitis cirrhosis, right pleural effusion, primary hepatocellular carcinoma of the left liver, hepatitis B virus carriage, and liver occupations. Complications that may result from these conditions include, but are not limited to, the following: ascites, gastrointestinal bleeding, hepatic encephalopathy, hepatorenal syndrome, hepatopulmonary syndrome, greater susceptibility to other viral or bacterial infections, easy fatigue, jaundice (yellowing of the skin, eyes, and urine), and conditions, and hypersplenism. Please note that the above information is for reference only, the specific treatment plan and the risk of complications need to be combined with the patient's specific situation and the doctor's professional advice. Patients are advised to seek prompt medical attention for professional diagnosis and treatment.)

From the answering results of Q1 in Table 7, the answering results of the three models do not differ greatly, and the reply information is more complete. From the results of Q2, the ChatGLM model only mechanically replies to the patient with the information queried from the knowledge graph, while the GEBERT and Ours models are able to further analyse the connections between the data, with the Ours response being more in line with natural language conventions. In Q3, there are two other query relationships with backward and forward associations: “Patient-Disease” and “Disease-Complication,” which require that after querying the disease information, we should call the knowledge graph again to query the complication corresponding to the disease, and the system is set up to complete the query only once. Therefore, ChatGLM only replies the information of the disease, and GEBERT model replies on the basis of this information, which is more in line with the habit of natural language. Ours model gives some examples of complications corresponding to the disease, which can be used as a reference to the patient, and the answer is more complete.

## 4 Discussion

This study developed a Chinese question-answering system for liver cancer based on a knowledge graph and large language model (LLM). A knowledge graph was constructed by integrating data from hospital electronic medical records and information from professional medical websites. The system employs ChatGLM's function-calling capability combined with the BERT model to achieve question parsing and accurate querying, with ChatGLM ultimately generating natural language responses. Experimental results demonstrate that the system achieves a question parsing accuracy of 92.34% and an overall accuracy of 74% in liver cancer-related multiple-choice tests, outperforming comparative models. Case studies further indicate that the system's responses are more comprehensive and clinically relevant.

The data utilized in this study primarily originates from electronic medical records of liver cancer patients at Zhujiang Hospital of Southern Medical University and public information from http://xywy.com. This dual-source approach ensures data consistency and reliability during the initial development phase. By integrating and analyzing large volumes of real clinical data and public medical information, the system is endowed with a robust foundation of medical knowledge and patient cases, enabling efficient identification and accurate answering of liver cancer-related questions.

Electronic medical records from different hospitals vary in structure, terminology standards, and data completeness, which may affect the accuracy of entity recognition when the system is applied across hospitals. In the future, we will further incorporate electronic medical records from multiple sources, continuously expand the data scale, and comprehensively enhance the model's adaptability to various types of data to ensure that the system can perform stably and excellently in more application scenarios.

Question parsing, as the core component of the system, relies on ChatGLM's function calls for entity extraction and BERT for intent recognition, both of which are tightly coupled with the entity-relationship schema of the knowledge graph. This deep integration offers two key advantages: first, multi-technical synergy enables information complementarity. ChatGLM's function calls ensure precise entity identification, while BERT facilitates rapid intent parsing, significantly enhancing the accuracy and efficiency of semantic understanding. Second, the structured nature of the knowledge graph allows parsing results to be directly mapped to a professional knowledge network, ensuring evidence-based answers and mitigating the “hallucination” issue common in LLM-generated responses.

However, this model also has two limitations. (1) The entity schema is restricted, only supporting predefined entity types, making it difficult to handle emerging concepts or complex composite entities, which affects query completeness. (2) Knowledge inference is insufficient; directly returning no results when knowledge is missing impairs usability. Future optimizations will include introducing a dynamic entity schema expansion module to adapt to new concepts, and designing a probabilistic inference mechanism to quantitatively annotate knowledge gaps, with clear prompts of uncertainty when limited inference is allowed.

System performance was validated using automated metrics and comparisons with baseline models. Additionally, the designed single-hop, multi-hop, and complex reasoning multiple-choice questions were reviewed by hepatobiliary surgeons to ensure clinical relevance (Heyi et al., 2023). However, clinical validation is incomplete, lacking comparisons with authoritative clinical practice guidelines and evaluations by clinicians. In terms of user research, only automated metrics were considered, with no empirical usability testing. Future efforts will refine the evaluation framework by quantitatively aligning with clinical guidelines and conducting user studies involving liver cancer patients and healthcare providers to collect feedback on the system's practicality, usability, and credibility.

The system framework exhibits cross-domain adaptability. To extend it to other fields, three core steps are required: first, knowledge graph reconstruction, replacing the liver cancer specific graph with a domain specific one; second, retraining the intent recognition model using domain specific questions to fine tune the BERT intent classifier for accurate domain specific intent parsing; third, adapting LLM function parameters by updating ChatGLM's function calling parameters and defining domain specific entities and their descriptions to ensure precise entity extraction. This modular design enables efficient cross-domain migration, offering intelligent question answering support for a broader range of clinical scenarios.

## 5 Conclusions

In this paper, a liver cancer Q&A system is designed to make full use of medical open resources and patient electronic medical records to provide data support for the Q&A system. In order to fully analyse the patient's intention to provide more effective answers, the deep combination of medical knowledge graph with LLMs is explored in the question parsing section by using LLMs to extract the entities in the question and BERT to identify the user's intention. The experimental results show that the liver cancer Q&A system with the introduction of knowledge graphs and LLMs is able to answer patients' questions more comprehensively and effectively.

The liver cancer Q&A system combining LLMs and knowledge graph is an exploratory project of combining LLMs and knowledge graph, and there are still many parts that deserve to be improved. The system currently only realizes the already use on the disease of liver cancer, and the application to other diseases and fields still needs to be further explored.

## Data Availability

The raw data supporting the conclusions of this article will be made available by the authors, without undue reservation.
